# Influence of Electrohydrodynamics on Drying Characteristics, Physicochemical Properties, and Texture Characteristics of Potato

**DOI:** 10.3390/foods14101752

**Published:** 2025-05-15

**Authors:** Liye Zhang, Changjiang Ding, Huina Xiong, Tian Tian, Lifeng Zhu, Yufan Dou

**Affiliations:** 1 College of Science, Inner Mongolia University of Technology, Hohhot 010051, China; 202210907023@imut.edu.cn (L.Z.); 202213403101@imut.edu.cn (H.X.); 202210907037@imut.edu.cn (T.T.); 202210106001@imut.edu.cn (L.Z.); 202210106036@imut.edu.cn (Y.D.); 2 College of Electric Power, Inner Mongolia University of Technology, Hohhot 010051, China

**Keywords:** electrohydrodynamics, potato drying, fourier infrared spectroscopy, SEM, low-field NMR, textural properties

## Abstract

In order to systematically study the drying characteristics, microstructure, and mechanical properties of potato in an electrohydrodynamic (EHD) system, this paper uses different discharge voltages for drying experiments. The results show that the discharge produces reactive nitrogen–oxygen particles, the intensity of which increases with increasing voltage. Under 0–30 kV, the higher the electric field, the faster the drying speed of the samples. The 30 kV group dried 40.5% faster than the control group. The EHD drying group had better color, shrinkage, rehydration capacity, and effective water diffusion coefficient. Rehydration capacity was positively correlated with electric field strength. EHD-treated potato flakes form a porous network structure and expose starch granules, as shown by scanning electron microscopy and infrared spectroscopy. Higher voltage results in a greater proportion of ordered protein structure. EHD drying retains more water than the control, with the best results at 30 kV, as shown by low-field nuclear magnetic resonance (NMR). Texture analysis showed that adhesion peaked in the 25 kV group, and the 15 kV group had the best Young’s modulus and the lowest fracture rate. This study provides a theoretical basis and experimental foundation for the application of EHD drying technology in potato drying and deep processing.

## 1. Introduction

The potato is the world’s third most significant food crop [1]. Potatoes are rich in carbohydrates, protein, vitamin B complex, and dietary fiber and other nutrients. Studies have shown that these nutrients are conducive to enhancing satiety, stabilizing energy supply, promoting gastrointestinal peristalsis, and preventing blood glucose fluctuation, elevated blood pressure, and edema [2]. China’s potato demand exceeded 18 million tons in 2022 and annual potato production and demand continue to rise. Along with the residents’ awareness of healthy diet to enhance nutrient intake, potato has attracted much attention. At present, the state vigorously promotes the strategy of potato as a staple food, and strives to extend the deep processing industry chain [3]. However, fresh potatoes contain moisture, which can lead to browning, rot, and deterioration if not properly addressed. Therefore, effective drying of fresh potatoes is imperative to maintain their quality and extend their shelf life.

Drying often refers to the use of thermal energy to vaporize the moisture in the material, so that the material can be easily processed, transported, stored, and used [4]. Evaluating drying operations mainly concerns dry product quality and economy of the operation. Among the traditional methods of potato preservation, cold storage and cellar storage dominate. These two methods are mainly used to maintain the freshness of potatoes by precisely regulating temperature, humidity, and ventilation conditions. In contrast, drying is not a traditional and commonly used preservation method, but it has unique application value in specific scenarios. Using hot air drying, vacuum freeze drying, and other technologies [5], potatoes can be processed into food products suitable for aerospace, emergency relief, and other special industry needs. According to [6] research, drying and preserving potatoes is receiving attention in both practical production and emerging research. New drying techniques, such as hybrid drying (including freeze drying, infrared drying, and oven drying), maximize the retention of potato nutrients and flavor. In addition, the further processing of dried products and the addition of functional ingredients to whole potato flour to make nutritionally fortified foods can meet diversified needs, further expanding the application prospects of potato drying and preservation. However, the existing drying technology has the problems of serious nutrition loss and high energy consumption. Therefore, it is of great strategic significance and practical value to explore new economic and green drying technologies to promote the transformation of potato from primary agricultural products to high value-added industrialized series products.

Electrohydrodynamics (EHD) technology constitutes a novel form of non-thermal drying technology that utilizes ion beams, the interaction of water molecules within the material, and external blowing to facilitate the drying process [7]. Within this technology, the electric field force directs the movement of water molecules within a liquid, thereby extracting water molecules from the surface layer of water [8]. In this process, the irregular movement of water molecules as a result of the action of the electrostatic force becomes directional movement in the direction of increasing electric field strength. The material temperature does not rise, which can make the effective components in the material without loss. This makes the technology especially suitable for the drying of heat-sensitive materials [9].

The utilization of EHD drying technology in potato drying has been the subject of preliminary research. Ding et al. [10] and other researchers have conducted a comparative analysis of high-voltage electric field drying and hot air drying, focusing on the effects of different drying materials, such as potatoes and carrots. The results of this analysis indicate that high-voltage electric field drying can accelerate the drying process, resulting in faster drying speeds compared to hot air drying. Additionally, the temperature during high-voltage electric field drying does not increase significantly, which is advantageous in preserving the nutrients present in the original material. Bao et al.’s [11] study of high-voltage electric field drying characteristics of potatoes revealed that an increase in voltage led to an increase in the average drying rate of potatoes and a decrease in the rehydration rate. Yang et al.’s [12] findings indicated that high-voltage electric fields exerted a lesser effect on the secondary structure of potato protein compared to hot air drying, contributing to a reduction in nutrient loss.

Most scholars are concerned with the optimization of EHD equipment and its effect on the drying characteristics of the material. There are few studies on the drying quality and internal moisture distribution of potato by EHD technology, and the effect on mechanical properties has not yet been studied. Therefore, the purposes of this study are: (1) to study the effect of different voltages on the drying quality of potato protein secondary structure and other drying qualities under EHD drying technology; (2) to investigate the distribution of moisture in the samples after drying at different voltages; (3) to elucidate the changes in the textural characteristics of potato under different voltages of EHD drying technology.

## 2. Materials and Methods

### 2.1. Experimental Material

The fresh, disease-free raw potato utilized in this experiment was procured from a supermarket located in proximity to Inner Mongolia University of Technology, Hohhot, Inner Mongolia. Fresh and disease-free potato raw materials were used in this experiment because diseased or spoiled potato may interfere with the drying process and nutrient retention due to microbial metabolism, nutrient degradation, and other factors. The samples were meticulously washed under running water and peeled, subsequently being cut into thin slices measuring 3 cm × 3 cm × 0.3 cm.

### 2.2. Experimental Setups

**Figure 1:**
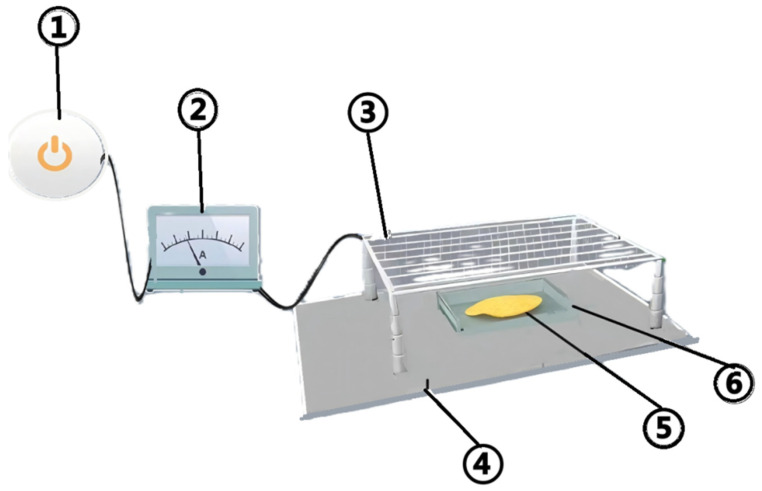

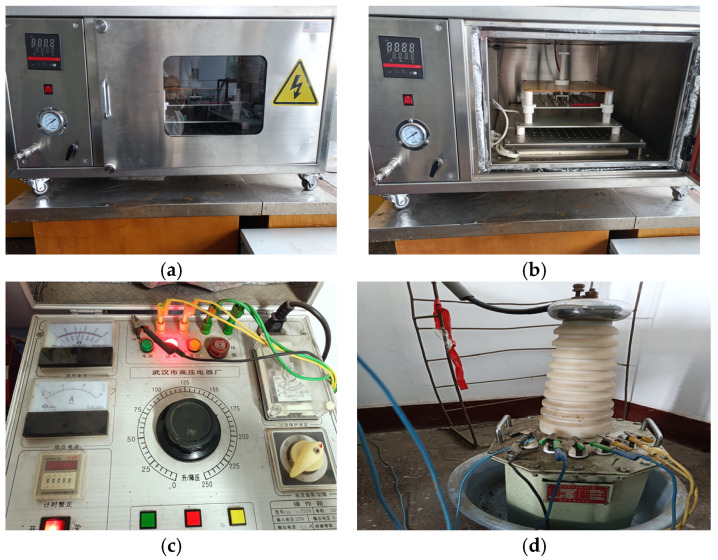
Structure of EHD drying equipment and actual drying equipment. 1. High-voltage power supply; 2. control system; 3. needle tip electrode; 4. grounding electrode; 5. potato slice; 6. 190 mm × 120 mm size Picasso Petri dishes. (**a**,**b**) Multi-needle-plate electrode system; (**c**) high-voltage power supplies; (**d**) controllers. (Figure (in English, meaning ‘diagram’ or ‘illustration’) is used throughout the manuscript to refer to the various visual representations).

The high-voltage electric field drying experimental device is illustrated in Figure 1. The device consists of a high-voltage power supply (YD(JZ)-1.5/50, Wuhan Boyu Power Equipment Co., Ltd., Wuhan, China), a controller (KZX-1.5 KVA, Wuhan), and a multi-needle-plate electrode system. The control system of the high-voltage power supply has the ability to adjust the AC output voltage within the range of 0–50 kV and the DC output voltage within the range of 0–70 kV separately. The upper electrode plate of the electrode system is a multi-needle electrode, which is connected to the AC voltage (the EHD drying rate and dehydration effect of the AC power supply is better than that of the DC power supply [13]). The configuration of the electrode system comprises multi-needle electrodes, with a distance of 40 mm between adjacent needles. The needles are 20 mm in length, 1 mm in diameter, and are separated by a distance of 100 mm from the lower electrode plate. The lower electrode is grounded. The lower electrode plate is composed of a stainless-steel plate of dimensions 1000 mm × 450 mm. The high-voltage electric field drying equipment used in this experiment has an installed power of 5.285 W, which can satisfy the energy consumption demand during the experiment [14]. Potato drying experiments were conducted at an ambient room heat of 24 ± 1 °C, and the air had a relative moisture content of 25 ± 1% RH.

### 2.3. Experimental Methods

In order to effectively inhibit the polyphenol oxidase (PPO) activity of potato when it is being dried [15], the experiment was conducted using a two-stage temperature-controlled treatment. In this treatment, the potato slices were first immersed in 100 °C ultrapure water (resistivity ≥ 18.2 MΩ-cm, in accordance with ASTM Type I) for 5 min of hot blanching, and then transferred to room temperature ultrapure water for 5 min of cooling, and the surface moisture was adsorbed by the filter paper for treatment [16]. Then, the potato was placed into the moisture rapid detector (Sh10A, Shanghai, China), and the initial wet basis of the potato was measured. The initial wet basis moisture content of the potato was 83.46 ± 1%.

Ultrapure water-treated potato slices were placed in plastic square dishes measuring 190 mm × 120 mm. The samples were then placed in an EHD drying system, with a mass ranging from 2.6 g to 2.9 g. In the pre-experiment, the drying effect in the range of 5–40 kV was tested, and it was found that overheating appeared on the surface of potato when the voltage exceeded 30 kV, while the improvement of drying efficiency was not significant when the voltage was lower than 15 kV. Yang et al.’s study on EHD drying of potatoes achieved good results with a voltage of 20 kV. Therefore, the present study optimized the voltage gradient on the basis of this study. Their voltages were 0 kV (control group), 15 kV, 20 kV, 25 kV, and 30 kV [12]. Six potato slices were retained at varying drying voltages to monitor the drying process and to measure parameters such as color difference, shrinkage, rehydration rate, and SEM. The mass of potato slices was measured every 0.5 h with the use of an electronic balance (BS124S, Shanghai Guanglu Electronic Technology Co., Ltd., Shanghai, China). Three independent measurements were taken for each group of samples and the final mass was expressed as mean ± standard deviation. The mass of dried potato was calculated to be 0.36–0.42 g with a standard deviation SD = 0.015 g, indicating that the drying was completed.

### 2.4. Measurement of Electric Field Discharge Characteristics

The discharge patterns at varying voltages were captured using a camera in a dark environment. The EHD discharge process at different voltages (0 kV, 15 kV, 20 kV, 25 kV, 30 kV) was processed using ICCD (DH334T-18 U-E3, ANDOR Technology Ltd., Belfast, UK) Plasma emission spectra in the 200–900 nm band range were obtained.

### 2.5. Moisture Content

The equations [17] for dry basis moisture content and moisture ratio of potato slices during drying were calculated as follows:

(1) $$M_{i}=\frac{m_{i}-m_{g}}{m_{g}}\times 100\%$$

(2) $$MR=\frac{M_{i}-M_{e}}{M_{0}-M_{e}}$$

where m_g_ is the dry mass (g) of the cylindrical potato, m_i_ is the mass (g) of the cylindrical potato when it is dried to i, M_i_ is the dry basis moisture content of the sample dried to time i, M_e_ is the dry base moisture content of the cylindrical potato when it is dried and balanced, M_o_ is the initial dry base moisture content of the cylindrical potato, and MR is the moisture ratio of potato slices; i stands for the ith moment in the experiment, and the unit is h (hour). The initial moment was set to 0.5 h according to the experiment conducted, and recordings were made at 0.5 h intervals.

### 2.6. Drying Rate

The drying rate of two neighboring potato slices at the same time interval is defined [17] as:

(3) $$DR=\frac{M_{t}-M_{t+\Delta t}}{\Delta t}$$

where DR is the drying rate (g water/g dry matter × h), M_t_ is the dry basis water content of cylindrical potato at the moment of t, and M_t+∆t_ is the dry basis water content of cylindrical potato at the moment of t + ∆t.

### 2.7. Effective Water Diffusion Coefficient

Fick’s second law is a useful tool for characterizing the drying process of biologics. When quantitative analysis of moisture transfer behavior during the drying of biomaterials is desired, the effective diffusion coefficient (D_eff_) can be modeled mathematically using this law. For a prolonged desiccation process and the value of MR is less than 0.6, the following equation [18] is applicable:

(4) $$\ln(MR)=-\frac{\pi^{2}D_{\mathrm{eff}}}{4L^{2}}t+\ln(\frac{8}{\pi^{2}})$$

where L is equal to half the thickness of the sample. When processing the data obtained in the experiment, it is necessary to fit the curve of ln (MR) versus drying time t by linear regression method, and its slope (K) and effective diffusion coefficient have the following quantitative relationship:

(5) $$D_{eff}=-\frac{4L^{2}K}{\pi^{2}}$$

### 2.8. Shrinkage Rate

In the experiment, the method of measurement employed was the quartz sand volume replacement method, and the incremental method was used to accurately weigh 40 g of dried quartz sand. The quartz sand was then transferred to a 100 mL graduated cylindrical container. Subsequently, 10 mL of quartz sand was filled at the bottom. Consequently, a piece of dried potato slice was placed vertically, and the quartz sand was gradually added to the container up to the 40 mL graduated scale. Finally, the mass of the remaining quartz sand was weighed. The mass of quartz sand was recorded, and three parallel experiments were carried out. The average value was calculated and regarded as the final result. Shrinkage, defined in terms of percentage reduction in the capacity of the dried potato relative to the volume it was at first, was calculated using the following formula [19]:

(6) $$SR=\frac{V_{0}-V_{f}}{V_{0}}\times 100\%$$

where SR is the shrinkage of cylindrical potato, V_0_ is the volume of fresh cylindrical potato (cm^3^), and V_f_ is the volume of dry cylindrical potato (cm^3^).

### 2.9. Rehydration Rate

A quantity of 6.2 g of dried cylindrical potato was placed in 100 mL of deionized water. The cylindrical potato was removed after 7 h of immersion in an electrically heated thermostatic water tank (DK-600BS, Changzhou Henglong Instrument Co., Ltd., Changzhou, China) at 37 °C. This temperature was selected to activate the water channel protein activity and to avoid high-temperature-induced apoptosis [20]. The excess water on the surface was removed with filter paper. The mass of the potato before and after rehydration was recorded on an electronic balance and subsequently calculated. The rehydration rate of the potato was defined [21] as follows:

(7) $$RR=\frac{m_{a}}{m_{b}}$$

where RR is the rehydration rate of cylindrical potato, m_a_ is the mass of cylindrical potato after rehydration (g), and m_b_ is the mass of cylindrical potato before rehydration (g).

### 2.10. Sample Color Difference

The present experiment employed the CIE Lab color space for the measurement of color. A fully automatic colorimeter (3nh - NR60CP, Shenzhen 3nh Technology Co., Ltd., Shenzhen, China) was utilized to measure the color of the samples, taking the color of the white plate as the reference standard. For a given sample, the experiment was repeated five times, and the mean value was taken as the experimental result. The parameters that were measured consisted of brightness (L), redness (a), and yellowness (b). The total color distinction (∆E), chromaticity value C, whiteness value Whiteness, and hue angle h° (0° and 360° for red, 270° for blue, 180° for green, and 90° for yellow) were calculated. Each parameter was calculated using the following equations [21]:

In the formula, L_0_, a_0_, and b_0_ represent the brightness, redness, and yellowness values of fresh cylindrical potato, while L_1_, a_1_, and b_1_ represent the corresponding values for dried cylindrical potato.

(8) $$\Delta E=\sqrt{{\left(L_{1}-L_{0}\right)}^{2}+{\left(a_{1}-a_{0}\right)}^{2}+{\left(b_{1}-b_{0}\right)}^{2}}$$

(9) $$c=\sqrt{a_{1}^{2}+b_{1}^{2}}$$

(10) $$\mathrm{Whiteness}=100-\sqrt{{(100-L_{1})}^{2}+a_{1}^{2}+b_{1}^{2}}$$

(11) $$h{}^{\circ}=\tan^{-1}\left(\frac{b_{1}}{a_{1}}\right)$$

### 2.11. Infrared Spectrum

The dried potato products were ground, mixed with potassium bromide to ensure uniform distribution, and sieved. After that, they were inserted into a tablet press (HY-12, Jiangyin Huayu Pharmaceutical Machinery Co., Ltd., Jiangyin, China) to carry out the tablet molding process. Afterward, the tablets underwent infrared scanning via an FTIR spectrometer (Nicolet iS10, Thermo Fisher Scientific, located in Waltham, Massachusetts, USA). The scanning occurred within a wavelength range of 400–4000 cm^−1^, with the spectrometer set at a resolution of 4 cm^−1^ and a signal-to-noise ratio of 50,000:1. The scanning was carried out 32 times consecutively to eliminate background interferences. This entire procedure was replicated 32 times to acquire the infrared scanning spectra of the samples.

### 2.12. Protein Secondary Structures

The amide I band in infrared spectroscopy is a critical region for analyzing the secondary structure of proteins. The absorption peaks in this region are characterized in order to quantitatively resolve the composition ratio of different secondary structures, including α-helix, β-sheet, β-turn, β-antiparallel, and random coil, in proteins.

### 2.13. Scanning Electron Microscope (SEM)

To observe the effects and changes of each treatment group on the microstructure of the potato surface, thin slices of dried potato were affixed to a sample stage with conductive tape and then sprayed with a thin layer of gold using an ion sputtering coater. The samples were subsequently loaded into a SU8020 scanning electron microscope (SEM) manufactured by Hitachi High-Tech Corporation, located in Tokyo, Japan. With an acceleration voltage of 5 kV, the SEM scanned the identical position on various samples, thus enabling the acquisition of SEM images.

### 2.14. Low-Field Nuclear Magnetic Resonance (LF-NMR)

A low-field nuclear magnetic resonance (LF-NMR) analyzer (MicroMR12-025V, Niu Buying Analytical Instruments Co., Ltd., Niu Buying, Suzhou, China) was utilized to assess the moisture status of potato samples subjected to different voltages. The parameters of the LF-NMR measurements are outlined below: resonance frequency SF = 12 MHz, the echo time (TE) was set to 0.16 milliseconds (ms), the relaxation decay time (TW) was 1000 milliseconds (ms), and the cumulative number (NS) was 32. The 90-degree pulse width was 7.32 microseconds (µs), and the 180-degree pulse width was 11.20 µs. The receiver bandwidth (SW) was set to 200 kilohertz (kHz). The accumulation number NS was set to 32, and the pulse width was 7.32 µs at 90 degrees and 11.20 µs at 180 degrees. The receiver bandwidth (SW) was set to 200 kHz. The dried potato samples were placed into a 1-inch OD NMR tube and tested using an NMR analyzer and transverse relaxation time (T_2_) using the Q-CPMG sequence.

### 2.15. Texture Properties

The texture profile analysis (TPA) (TA.XT PlusC, Stable Micro Systems, Godalming, UK) was utilized to ascertain the textural properties of potato samples subjected to different voltages during the drying process. The tool used in the test is a flat-bottomed cylindrical probe P/50 with a diameter of 50 mm. TPA, an acronym for “texture profile analysis,” is a method that primarily emulates the biting action of the human mouth. Consequently, it is referred to as the “twice-chewing test” [22]. A key benefit of the TPA analysis method is that it can measure a variety of textural parameters, such as hardness, adhesion, elasticity, cohesion, and chewiness, all in a single experiment. In comparison to sensory analysis, the TPA method offers the advantage of providing more objective, efficient, and consistent assessments of food product quality. For example, hardness is determined by recording the force value at the target displacement during the first cycle; elasticity is observed by examining the curve between two compressions, with good elasticity samples showing a gradual decrease in force value and a rebound before the second compression, while poor elasticity samples show a rapid decrease in force value without rebound; cohesion is related to the area under the curve, and the ratio of the area under the second compression curve to that of the first compression curve being closer to 1 indicates better cohesion. By combining force and displacement data, the internal bonding strength of the sample can be comprehensively analyzed, and samples with high cohesion have a tightly bonded internal structure that is less prone to dispersion. Each set of tests was repeated three times to ensure reliability and accuracy of the results. The initial conditions for determining the surface viscosity of potato tablets were as follows: test speed 30 mm/s, trigger force 1.5 g, and deformation rate 70%.

### 2.16. Statistical Analysis

Each experimental series was replicated thrice, and the resulting data were presented as the average ± standard deviation. The differences in the data concerning the moisture content, drying rate, color difference, shrinkage, and mechanical properties of the potato were analyzed using one-way ANOVA (one-way ANOVA). Statistical significance was considered to have been achieved at *p* < 0.05. Graphs were plotted using relevant software.

## 3. Results and Analysis

### 3.1. Discharge Characteristics of EHD Systems

**Figure 2:**
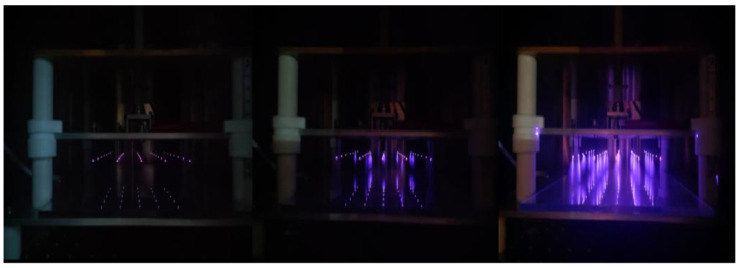
Discharge morphology under various drying voltages.

As illustrated in Figure 2, the morphological characteristics of corona discharge (EHD) vary with different voltages. Empirical evidence demonstrates that the discharge process is accompanied by a blue–violet glow phenomenon in a dark environment. Notably, the brightness of the glow exhibits a substantial voltage dependence, i.e., an increase in applied voltage results in a gradual enhancement of the intensity and color saturation of the glow. This phenomenon intuitively reflects the positive correlation between the discharge energy and the voltage parameter, indicating that higher voltage input can effectively improve the discharge efficiency and visualization effect of the EHD system.

**Figure 3:**
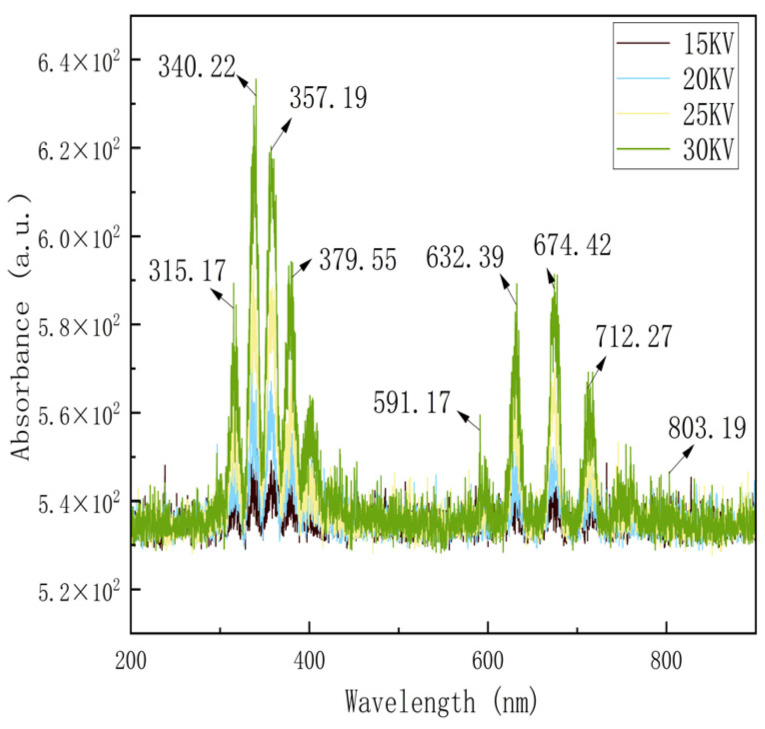
Emission spectra at different drying voltages.

The analysis of the emission spectra presented in Figure 3 suggests that when a high-voltage is applied to the needle-plate electrode of the EHD dryer, a corona discharge occurs at the point of the needle electrode, leading to the ionization of the surrounding air into a plasma containing free radicals and reactive atoms. The spectral data analysis reveals that the plasma emission spectrum predominantly exhibits the characteristic spectral lines of oxygen and nitrogen-derived reactive species within the 200–900 nanometer band range. The formation mechanism of this spectral feature is closely related to the voltage parameter; an increase in applied voltage results in an increase in the high-energy electron density in the plasma, which prompts more nitrogen molecules to be excited and ionized, thereby enhancing the generation efficiency of RONS. As can be seen from Figure 3, the concentration of RONS increases with the increase in voltage. Among them, N_2_* appeared at the strong peak positions near 315.17 nm, 340.22 nm, 357.19 nm, 379.55 nm, and under 591.17 nm, which is in the first positive band system. The oxygen atom O appeared at 632.39 nm, 674.42 nm, 591.17 nm, 712.27 nm, and 803.19 nm. The presence of iron and copper in the plasma generated by corona discharge is a possibility [23]. It has been shown that in corona discharge drying [24], the active nitrogen–oxygen species (H_2_O_2_, O_3_, and NO) are considered to be distributed roughly uniformly above the flat plate electrode at the same instant in time, with the exception of the area below the needle tip, where the concentration of H_2_O_2_, O_3_, and NO tends to increase cumulatively with time. However, in the present study, reactive nitrogen was found to constitute the bulk of the oxidizing active component. The aforementioned conclusions offer direct spectroscopic evidence to facilitate a comprehensive understanding of the oxidation reaction occurring on the surface of the material during the EHD drying process. Furthermore, these findings provide a theoretical groundwork for optimizing the discharge parameters to enhance the drying productivity.

### 3.2. Drying Characteristics

#### 3.2.1. Drying Moisture Content, Drying Rate, and Drying Time Analysis

**Figure 4:**
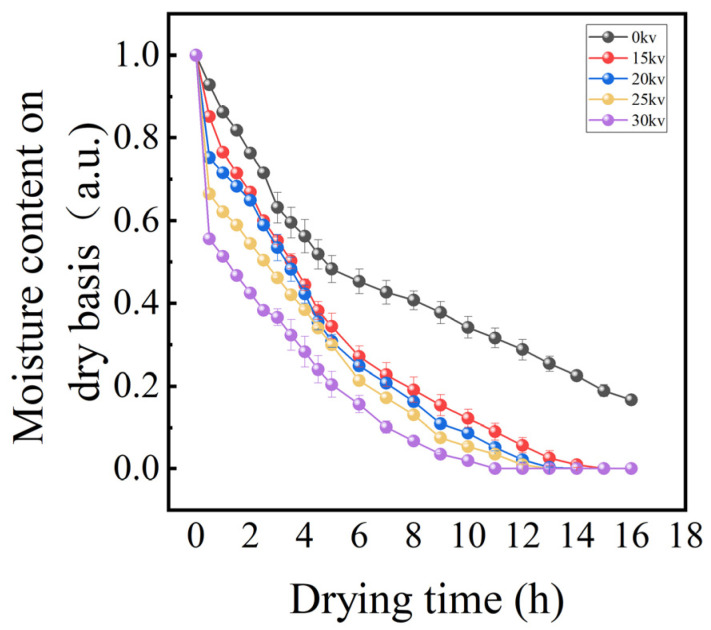
Moisture content variation of potato samples over time under different drying voltages.

**Figure 5:**
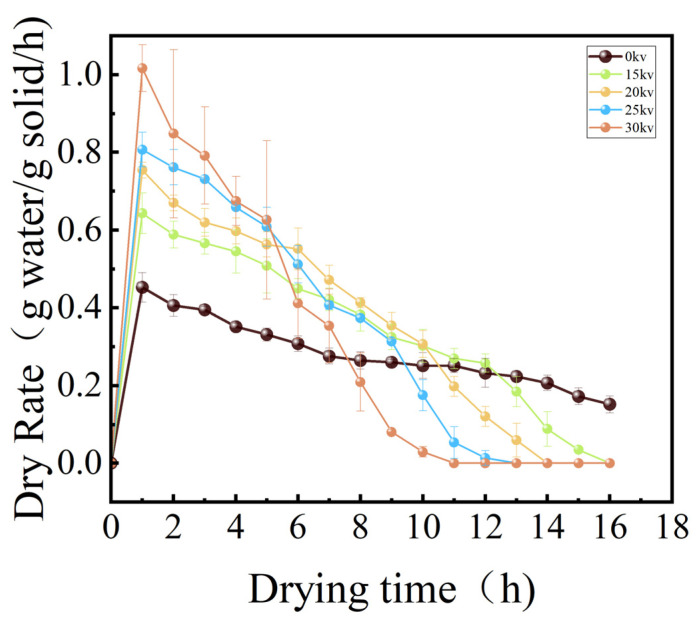
Drying rate versus time curve of potato samples under different drying voltage conditions.

As illustrated in Figure 4, the dynamic change curves of moisture content in potato samples with drying time under different drying voltage conditions are presented. The results of the analysis of variance (ANOVA) showed (*p* < 0.05) that there were significant differences in the drying characteristics of the potato under different drying voltage conditions. Specifically, in the initial stage of drying (0–30 min), the forced convection effect induced by electrostatic wind rapidly vaporized the free water covering the potato, while the internal water migrated to the surface driven by the vapor pressure gradient, forming a typical constant velocity drying stage. As the drying process progresses (30–120 min), when the surface water diffusion rate surpasses the internal water migration rate, the material enters the reduced-rate drying stage, during which time the internal water transport is progressively impeded, and the rate of moisture content decrease slows down with the reduction of the moisture gradient [25]. Figure 5 demonstrates the correlation between drying rate and voltage parameters in the EHD drying treatment. The experimental data demonstrate a proportional relationship between drying rate and voltage inside a specific range, compatible with the findings of EHD drying studies of tomato by Esehaghbeygi et al. [26]. Specifically, voltage enhancement significantly accelerates material surface moisture vaporization and internal moisture migration by enhancing the ionic wind effect and corona discharge intensity. Among them, the 0 kV control group showed typical natural convection drying characteristics with the longest drying cycle, while the EHD treatment group shortened the drying time to 40.5% (i.e., 2.47-fold enhancement) of that of the control group at 30 kV, and still achieved a 1.73-fold efficiency enhancement at 15 kV. Also due to the ionic wind effect in EHD by enhancing surface convective heat transfer and internal moisture migration, we observed a significant reduction in drying cycle time using EHD drying technology, which provides a theoretical basis for efficient dehydration of food materials.

**Figure 6:**
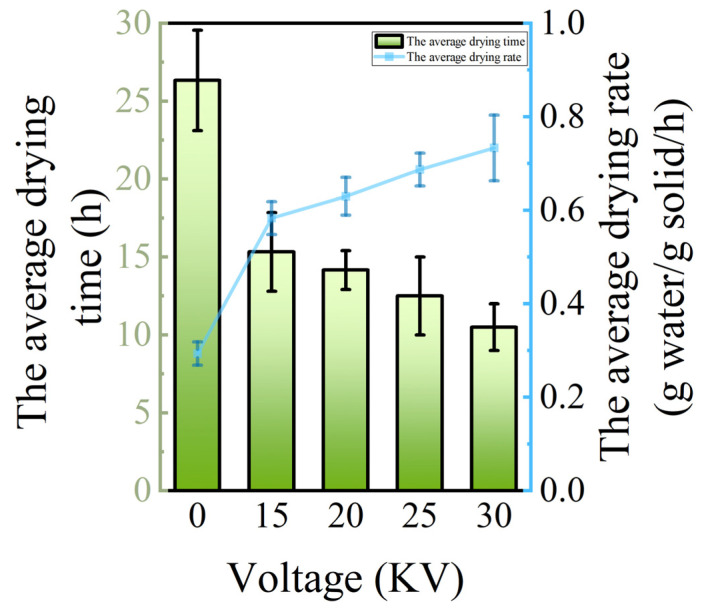
Change curves of average drying time and average drying rate of potato samples under different drying voltage conditions.

The joint analysis of Figure 4, Figure 5 and Figure 6 showed that different voltage parameters significantly affected the potato drying kinetics under EHD drying treatment. From the moisture content–time curve characteristics, the moisture content of potato decreased exponentially with the increase in drying time. In the early stage of drying, the moisture content ratio of potato decreased rapidly, and with the prolongation of time, the rate of decrease gradually slowed down. The fastest decrease in moisture content ratio was 30 kV.

**Figure 7:**
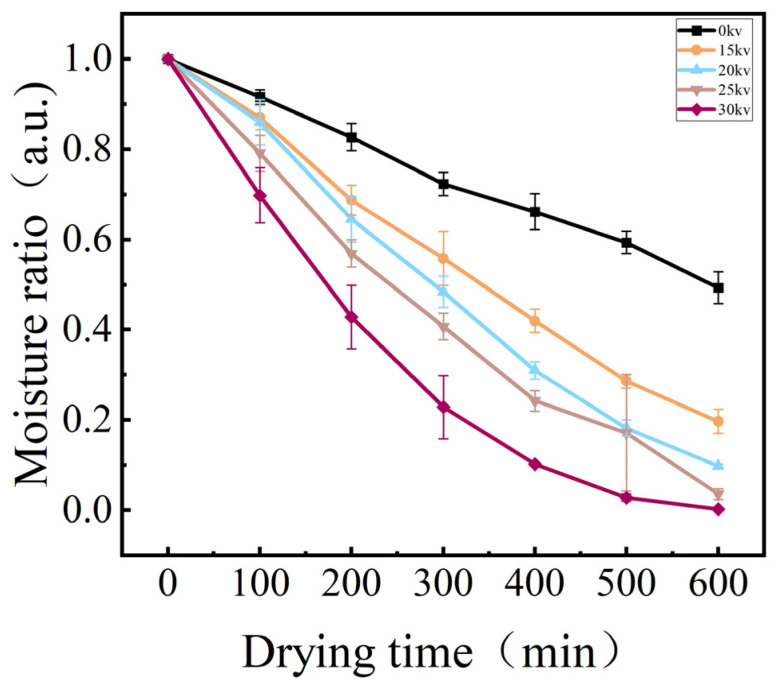
Moisture ratio of potato samples under different drying voltage conditions.

The moisture ratio (MR) is a pivotal kinetic parameter for characterizing the drying process of materials, with its dynamic changes offering direct insights into the drying efficiency and the underlying quality formation mechanism. In the electrohydrodynamic drying of Lycium barbarum, Yang et al. [14] observed that the strength of the electric field plays a substantial role in regulating the rate of material dehydration. A comprehensive analysis of the experimental results, as depicted in Figure 4 and Figure 7, reveals a substantial decrease in the water content of potato samples subjected to EHD, a finding that aligns with the conclusions drawn by Yang et al. Subsequent analysis revealed a distinctive voltage dependence in the enhanced dewatering process. The moisture ratio exhibited a rapid decline in response to an increase in voltage, with higher voltages resulting in faster reduction.

From the perspective of the drying rate–moisture content correlation, the accelerated decrease in moisture content and the increase in drying rate of the potato in the EHD treatment group can be directly attributed to the enhanced effect of ionized air. It is worth noting that 0 kV shows some increase in the DR value of the drying rate after ten hours of drying, which is due to the fact that there was an increase in the velocity of the airflow in the experiments, which led to an increase in the drying rate. This experiment utilizes the property of non-significant warming during electrohydrodynamic drying to achieve temperature regulation. This technique accelerates the drying process by driving the fluid motion through the electric field force, and the main energy is used to promote the evaporation of the liquid rather than converting it into thermal energy to raise the temperature. At the same time, this feature does not cause a significant increase in temperature with the increase in voltage, which provides a guarantee for accurate temperature control, and this low-temperature feature is important for the retention of heat-sensitive components [27]. This low-temperature characteristic is crucial for preserving heat-sensitive components. The outcomes align with those reported by Dinani et al. [28] in the context of apple slices drying, validating the synergistic enhancement of drying efficiency and quality through the modulation of electric field parameters.

#### 3.2.2. Analysis of Effective Water Diffusion Coefficient

The effective moisture diffusion coefficient is a core kinetic parameter for drying fruits and vegetables (see Table 1). As shown therein, the effective water diffusion coefficients ranged from 1.5456 × 10^−10^ to 1.7417 × 10^−11^ m^²^/s under different voltage conditions during EHD drying of the potato. These coefficients were significantly higher than those of the control group in all treatment groups. This phenomenon was attributed to the electrohydrodynamics-induced electroporation effect, which is defined as the formation of microporous channels by disrupting the integrity of the cell membrane. This, in turn, enhanced the cell permeability 3–5-fold, thus facilitating water migration. This result is consistent with the experimental findings of Li et al. [29], who used low-temperature plasma to treat corn kernels.

In addition to the analysis of the effective water diffusion coefficient (D_eff_), we introduced the statistical parameters F-value, *p*-value, and goodness-of-fit R^2^ to evaluate the regression models under different voltage conditions. F-value was used to test the overall significance of the regression equation, and to measure the combined effect of the independent variables on the dependent variable by comparing the model variance to the residual variance, with a larger F-value resulting in a more explanatory model. *p*-value is the probability of obtaining a significant linear relationship between the independent variables and the dependent variable when the original hypothesis (the probability of obtaining the current or more extreme result when the independent variable is not linearly related to the dependent variable) is true, with *p* < 0.05 indicating a significant linear relationship between the variables. R^2^, on the other hand, is used to measure the goodness-of-fit of the regression straight line to the observed data, and the closer its value is to 1, the better the model explains the data. In this study, the F-values under different voltage conditions are all in the range of 120.37–130.76, and the *p* values are all much less than 0.05, which indicates that the regression model as a whole is significantly valid under different voltage conditions, and that the independent variables have a significant explanatory power for the dependent variables. The goodness-of-fit is measured by R^2^, which fluctuates from 0.84784 to 0.98958 in this study, indicating a better model fit. There is a strong link between F-value, *p*-value, and R^2^. When the voltage is 0 kV and 15 kV, the F and *p* values prove that the model is significant and R^2^ is close to 1, indicating that the model is not only statistically significant but also highly able to fit the data; at 20 kV, although R^2^ decreases, the F and *p* values show that the model is significant as a whole, indicating that the model has validity even though the degree of fit is slightly reduced; whereas, at 25 kV and 30 kV, the F and *p* values ensure that the model is significant. The decrease in R^2^ suggests that the model has some limitations in fitting the data. Therefore, the combination of F-value, *p*-value, and R^2^ enables a comprehensive assessment of the significance, validity, and quality of fit of the regression model at different voltages.

It is noteworthy that the elevated electric field intensity exhibits a dual effect: on the one hand, it strengthens the degree of cell membrane damage, which significantly increases the water diffusion coefficient; on the other hand, it exacerbates the treatment inhomogeneity (e.g., the difference in the degree of cellular damage in different regions), which leads to a decrease in the value of R^2^, indicating the enhancement of the nonlinear effect. This contradictory relationship between treatment efficiency and data fit provides a theoretical basis for optimizing the electric field parameters.

### 3.3. Quality Analysis

#### 3.3.1. Color Analytics

Color, a pivotal component of food quality assessment, exerts a substantial influence on the sensory quality of dried potato chips. During the drying process of dehydrated vegetables, chemical reactions, including the Maillard reaction, pigment degradation, enzymatic browning, and vitamin C oxidation, significantly impact the color and flavor of the final product. Polyphenolic compounds present in the material also significantly influence the color and flavor of the treated tissue [30]. The yellowness, redness, and brightness of the drying endpoints are of particular significance. Low redness and high brightness values generally indicate superior color performance, while the merits and drawbacks of the yellowness values require scientific evaluation within the context of specific application scenarios. The utilization of high yellowness varieties for nutritional enhancement and low yellowness varieties for culinary color homogeneity is recommended.

As illustrated in Table 2, the experimental data demonstrate that the use of EHD electric field drying substantially improves the brightness of potato crisps, with an increase ranging from 45.86% to 56.92%, compared to the 0 kV control group. This enhancement is accompanied by an increase in whiteness, which is crucial for the industrial production of high-quality dehydrated vegetables, meeting the standard for optimal whiteness. The redness index exhibited distinct characteristics: the redness value of the 15 kV voltage treatment group increased by 15.52%, while the redness value of the 20–30 kV voltage treatment group decreased by 3.45% to 16.9% (enzymatic reaction). Notably, the yellowness value of each voltage treatment group increased more than twofold, indicating a significant enhancement in the yellow hue. On the one hand, the significant increase in yellowness counteracts the fluctuation of redness, resulting in the formation of highly saturated color values dominated by yellow (C). On the other hand, the significant increase in yellowness causes the hue angle (h°) to converge from the red–yellow mixture of the control group (h° ≈ 80°) to pure yellow (h° = 90°) (enzymatic reaction). Bai et al. [31] showed that EHD drying was effective in preserving the natural color of seaweeds, giving them an olive color, and that the EHD-dried seaweeds had a more lustrous appearance and less deformation compared to the oven-dried samples. This is consistent with the conclusion of this study that the samples in the EHD-dried group exhibited high yellowness and low redness compared to the control group. This color profile, characterized by its high brightness, low redness, and high yellowness, is indicative of enhanced nutrient retention and meets the quality requirements for golden yellow coloration in baked goods. The study demonstrates that within the voltage range of 20–30 kV, EHD treatment ensures optimal drying efficiency while achieving the desired color regulation, resulting in visual characteristics of “high brightness yellow, low reddish brown”. This provides substantial technical support for the industrial production of high-quality dried potato chips.

#### 3.3.2. Volume Shrinkage

**Figure 8:**
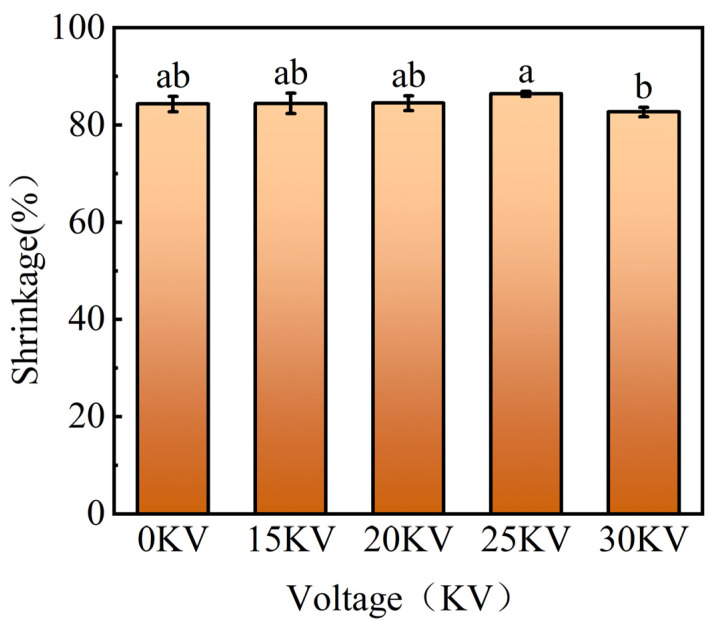
Variation of shrinkage of potato samples under different drying voltage conditions. Different letters indicate significant differences between sample means (*p* < 0.05).

The results of Figure 8 show that the contraction rates of 0, 15, 20, 25, and 30 kV drying groups were 84.31%, 84.48%, 84.49%, 86.41%, and 82.67%. Except for the 30 kV group, the shrinkage rate of the control group was slightly lower than that of the EHD drying group. The EHD drying group exhibited superior surface smoothness while maintaining a higher shrinkage rate. Shrinkage, a core index for evaluating drying quality, is primarily influenced by the inherent structural properties of materials and the final moisture content [32]. The impact of varying voltage parameters on shrinkage in this study did not demonstrate statistical significance, aligning with the findings of electrohydrodynamic drying of mushrooms by Xiao et al. [33]. Conclusively, these cross-material findings validate the unique advantages of EHD drying technology in maintaining the structural integrity of materials.

#### 3.3.3. Rehydration Performance Analysis

**Figure 9:**
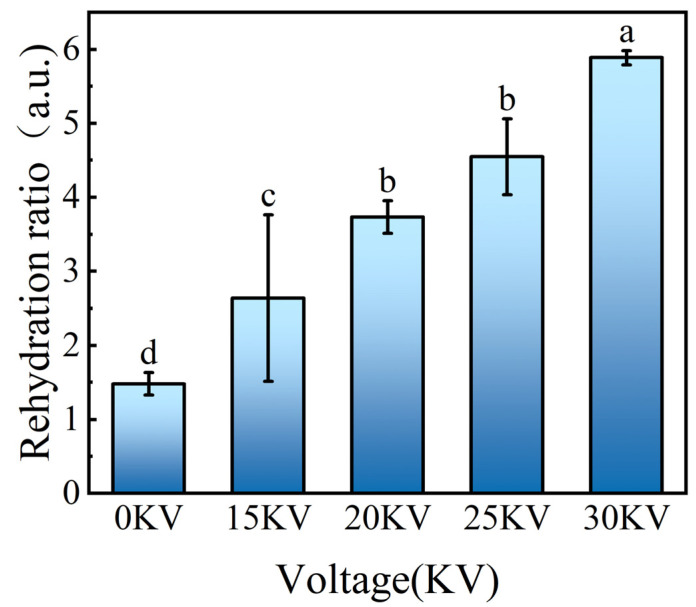
Rehydration performance of potato samples under different drying voltage conditions. Different letters indicate significant differences between sample means (*p* < 0.05).

As illustrated in Figure 9, the rehydration rates of the 0, 15, 20, 25, and 30 kV drying groups were 1.48, 2.64, 3.73, 4.55, and 5.89, respectively. Statistical analyses indicated that the rehydration rate of the EHD drying group was significantly higher than that of the control group, and showed an obvious voltage gradient dependence. This performance enhancement was closely related to the internal structural integrity of the material. Scanning electron microscopy analysis demonstrated that the samples within the EHD-treated group maintained a more intact cellular structure and higher porosity, resulting in more effective water transport channels. The results of the analysis of variance (ANOVA) further confirmed that the drying voltage was a key factor influencing the rehydration rate. Esehaghbeygi et al. [34] also found in a banana slice study that the rehydration rate was enhanced in the 10 kV treatment group, which was attributed to the non-thermal effect of electrohydrodynamic drying (treatment temperature ≤ 45 °C) effectively inhibiting the thermal degradation of cell wall polysaccharides. In this study, thermodynamic analysis revealed that the non-thermal properties of EHD drying enabled the dried samples to exhibit faster water absorption kinetics and higher water holding capacity during rehydration.

### 3.4. Infrared Spectral Analysis

**Figure 10:**
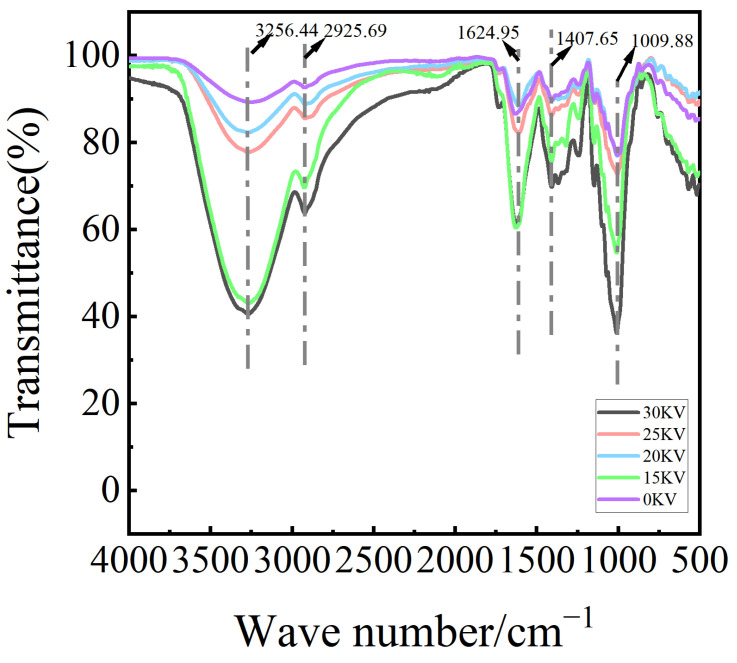
Infrared spectroscopic potato samples at different drying voltage conditions.

Infrared spectroscopy has been demonstrated to reveal the presence of characteristic vibrational absorption peaks in molecules, attributable to the interaction of infrared light with specific molecular structures. This phenomenon, governed by the principle of molecular structure, has been found to exhibit a strict correspondence with the location and intensity of absorption peaks. The application of infrared spectroscopy facilitates the identification and analysis of functional groups, both qualitatively and quantitatively [35]. The analysis of infrared spectra of potatoes subjected to varying drying voltages has been shown to exhibit significant regular changes in the infrared spectral characteristics. As illustrated in Figure 10, the infrared absorption crests of each group of samples were highly coincident, indicating that the molecular skeleton structure remained stable, but the intensity of the characteristic peaks showed obvious differences. Specifically, the transmittance exhibited a three-stage trend with increasing voltage, in which the peak intensity of the 30 kV treatment group reached the maximum, and the peak intensities of all EHD treatment groups were significantly higher than those of the 0 kV control group.

In terms of characteristic functional group analysis, the potato exhibited strong absorption bands at 3256.44 cm^−1^ and 2925.69 cm^−1^, which corresponded to the characteristic O-H and C-H telescoping vibrations, respectively [33]. The peaks observed at 1624.95 cm^−1^ were attributed to the carboxyl group or the C=O stretches of esters, which play a pivotal role in preserving the stability of the plant cell wall structure [36]. It is noteworthy that the positions of the absorption peaks at 1407.65 cm^−1^ (polysaccharide C-H bending vibration) and 1009.88 cm^−1^ (C-C single-bond stretching vibration) remained approximately constant across all treatment groups [37]. This observation suggests that the EHD drying process did not induce significant alterations in the fundamental structure of polysaccharides within potato.

The findings of this study demonstrate a high degree of congruence with the outcomes of electrohydrodynamic drying (EHD) studies on mushrooms by Xiao et al. [33] and on garlic slices by Han et al. [38]. Collectively, these studies substantiate the efficacy of EHD drying technology in preserving the primary components of agricultural products.

### 3.5. Protein Secondary Structure

The potato (Solanum tuberosum) is a tuberous plant that is a member of the nightshade family (Solanaceae). It contains approximately 1.6 to 2.1 g of protein per 100 g of edible portion. Proteins are macromolecules whose secondary structure determines their properties [39]. The amide I spectral analysis was conducted within the spectral range of 1600–1700 cm^−1^. Following baseline adjustment, Gaussian inverse convolution, second-order derivatives, and curve fitting, the correspondence between the subpeaks and the secondary structure will be obtained as follows. The following assignments are made: 1610–1642 cm^−1^ is β-sheet, 1642–1650 cm^−1^ is Random coil, 1650–1660 cm^−1^ is α-helix, the scope of 1660–1680 cm^−1^ corresponds to β-turn structures, while the range of 1680–1700 cm^−1^ aligns with β-antiparallel structures [40]. The α-helix is a common form of protein secondary structure, which is formed by the main chain of polypeptide chain around the central axis to form a right-handed helical conformation, every 3.6 amino acid residues helix up a circle, the pitch of the pitch is 0.54 nm. Its stable and orderly structure is mainly dependent on the peptide chain of the amino acid residues between the formation of hydrogen bonds [41]. α-helix and β-sheet structures are considered examples of ordered structures, while β-turn and random coil structures represent disordered structures [42]. As shown in Table 3, the analysis of the secondary structure of proteins in the potato indicates that β-sheet and β-turn structures are the main components of the protein’s secondary structure. The proportion of disordered structures identified in both the control and experimental groups (the range of voltages extends from low to high) were 42.26%, 35%, 36.13%, 35.41%, and 27.83%, respectively. Conversely, the percentage of ordered structures was 57.74%, 65%, 63.87%, 64.59%, and 72.17%, respectively. During EHD treatment, the proteomolecule underwent structural changes because of the under attack of energetic particles. The secondary structure of potato protein was found to be 52.41% α-helix and 28.71% irregularly coiled, indicating that the ordered structure (α-helix) was the predominant component in its natural state. This structural feature may be closely related to its function of catalyzing the synthesis of glycoside alkaloids, and the stability of the ordered structure helps to maintain the precise conformation of the enzyme active center [43]. The present study demonstrated that EHD drying exerts a substantial influence on the secondary structure of proteins. It was observed that an increase in voltage results in a decrease in the proportion of disordered structure and an increase in the proportion of ordered structure. This finding suggests that the gradual increase in the voltage parameter, as implemented in the present experiment, may contribute to the minimization of nutrient loss.

### 3.6. Microstructure Analysis

The high-voltage electric field induces the formation of nanoscale micropores in the cell membrane through the electroporation effect, which ultimately leads to the localized rupture of the cell membrane and the outflow of intracellular substances (e.g., water, electrolytes, etc.) [44]. At the same time, the electric field leads to a change in cell membrane permeability, which in turn causes cell deformation [45]. This was manifested in this experiment as enlarged cell gaps in low magnification SEM images. These physical changes acted synergistically to improve the potato drying rate.

**Figure 11:**
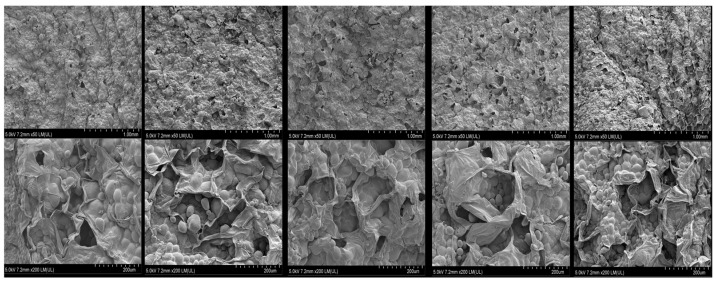
Surface microstructure of potato samples under different drying voltage conditions. (From left to right, these images correspond to voltages of 0 kV, 15 kV, 20 kV, 25 kV and 30 kV).

As demonstrated in Figure 11, the potato cells in the control group exhibited structural integrity, with discernible cell wall contours, and the starch granules were more effectively encapsulated within the cells, displaying a dense crystal arrangement. In contrast, EHD-treated potato demonstrated a directional fracture of the fiber structure, resulting in complete exposure of the starch granules due to disruption of the cell wall, and the surface evolved into a porous state. In low-intensity electric fields (15–30 kV), the samples exhibited predominant cell membrane perforation, rupture of cellular morphology, and ovoid-shaped starch granules. These observations are consistent with the findings of Nyssanbek et al. [46] in the plasma treatment of natural fibers, confirming that electric field-induced structural modification can significantly change the interfacial properties of vegetable materials. In the case of potato, the use of EHD drying technology has been shown to enhance the absorption of dietary fiber and improve the suitability of instant food processing for healthy individuals, while retaining the main nutrients of potato.

### 3.7. Low-Field NMR Analysis

Low-field nuclear magnetic resonance techniques, particularly the ^1^HLF-NMR, offer a distinctive approach for the visualization and analysis of food moisture dynamics. This method is characterized by its rapidity, sensitivity, non-destructive nature, and precision [47]. ^1^HLF-NMR refers to the High-field Low-frequency Nuclear Magnetic Resonance (HLF-NMR) technique. This is a technique with specific applications in the field of nuclear magnetic resonance, which utilizes a high magnetic field strength and a low radio frequency to analyze samples. It is based on the principle that by applying a specific magnetic field and RF pulses, the nuclei in the sample undergo resonance excursions to obtain information about the molecular structure and dynamics of the sample. In this study, we used the high-field low-frequency NMR technique to investigate the diffusion behavior and interactions of water molecules in dried potato. The LF-NMR detection principle underpins the technique’s ability to discern variations in the chemical microenvironment surrounding the hydrogen nucleus. These variations manifest as alterations in relaxation behavior, as evidenced by a shortened T_2_ relaxation time and a leftward shift in the peak spectra. This shift reflects the physical state of the hydrogen nucleus, where the degree of confinement is augmented or the degree of freedom is diminished [48].

**Figure 12:**
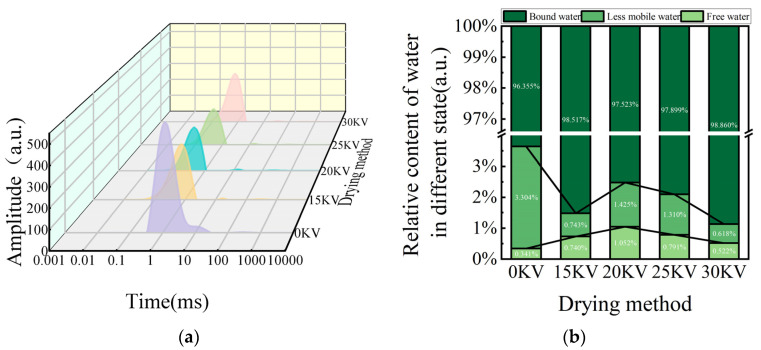
Low-field nuclear magnetic resonance (NMR) maps. (**a**) Transverse relaxation time (T_2_) spectrum; (**b**) moisture percentage.

An analysis of the T_2_ relaxation spectra in Figure 12a reveals that the potato samples subjected to varying drying conditions exhibit three distinct characteristic peaks: bound water (T_21_, 0.1–1 ms), not readily flowable water (T_22_, 1–10 ms), and free water (T_23_, 10–1000 ms), starting from the left [49]. The T_21_ peak corresponds to bound water forming strong hydrogen bonds with cell wall polysaccharides, starch, and other biomolecules [50], and it has the shortest relaxation time. The T_22_ peak represents immobile water confined by the structure of biofilm systems or fibrous networks, which is susceptible to phase transitions under specific conditions [51]. The T_23_ peak is attributed to free water in the interstitial space of the cell or in the vesicles, which has a long relaxation time originating from the solute rapid chemical exchange of molecules [52]. It is noteworthy that the area of each peak was significantly and positively correlated with the corresponding water content [49]. A comparative analysis of the EHD drying group and the control group (see Figure 11) reveals that in the T_2_ spectra of the EHD-treated group a lateral shift was observed. This finding suggests that this drying mode enhances the interaction between moisture and the solid matrix through ionic-induced effects. This phenomenon is highly consistent with the findings of Han et al. [38] in garlic drying, confirming the regulatory mechanism of electric field on moisture migration.

A subsequent analysis of the moisture percentage data in Figure 12b revealed that the percentage of bound water remained constant at approximately 98% for all EHD treatment groups. This finding corroborates the findings reported in the literature on the dehydration behaviors of materials such as blueberries [53] and lotus seeds [54], thereby providing evidence of the stability of the binding of biomolecules to water during the drying process.

Voltage gradient experiments revealed a unique mechanism of action of EHD drying: as the voltage increased, the bound water content exhibited an initial decrease followed by an increase, while the free water content exhibited an inverse change. The formation of this dynamic equilibrium may be related to the electric field-induced water redistribution, whereby moderate voltage (≤30 kV) promotes the conversion of free water to bound water by strengthening the interfacial polarization. The effectiveness of this mechanism in the inhibition of microbial growth by reducing moisture activity has been demonstrated in the drying of materials [55] such as apple [56] and lotus seed [57]. The results of the present study suggest that electrohydrodynamic drying can improve the mobility of free water inside potato slices and reduce the binding force of the tissue to water that is not easily flowing, thus promoting the migration of internal water to bound water.

### 3.8. Texture Properties Analysis

In recent years, texture profile analysis (TPA) techniques have seen a surge in utilization within the domain of food science. TPA serves as a pivotal indicator of quality deterioration during the drying process of solid foods. It has emerged as a crucial method for quantitatively assessing the mechanical properties of materials, exhibiting higher objectivity and reproducibility compared to sensory evaluation [58]. In this study, the effects of different drying processes on the structural properties of dried potato were analyzed by using the TPA puncture test system. This analysis revealed the structural evolution during drying through the parameters of hardness, elasticity, and chewiness.

**Figure 13:**
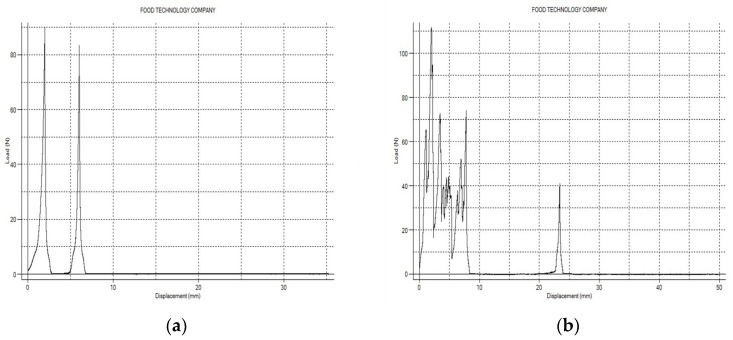

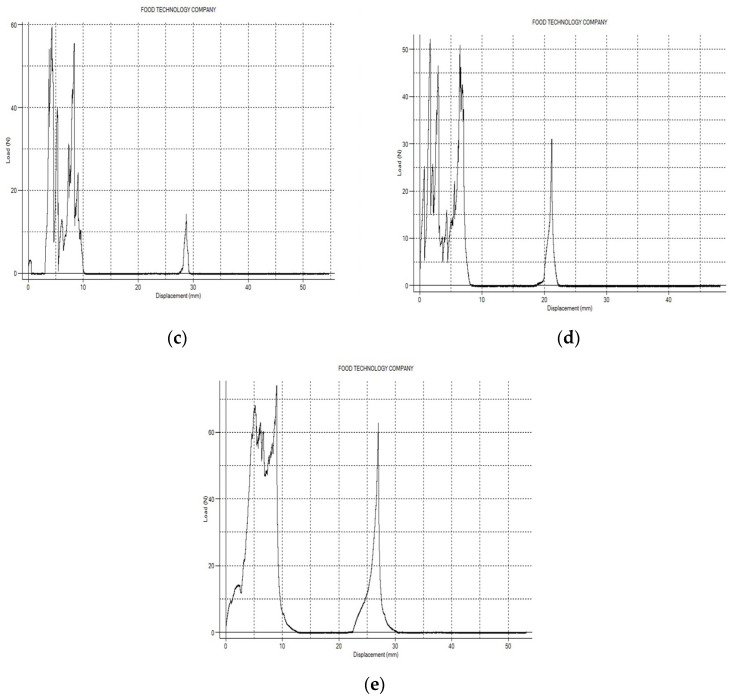
(**a**–**e**) Textural composition of potato samples at different drying voltages of 0 kV, 15 kV, 20 kV, 25 kV, and 30 kV, respectively.

In Figure 13a–e, the horizontal coordinate “Displacement (mm)” represents the distance traveled by the mass spectrometer probe, which is used to measure the progress of the test, and the vertical coordinate “Load (N)” represents the reaction force applied to the probe, which reflects the ability of the sample to resist the probe’s action. Figure 13a shows the texture test curve of the potato at 0 kV voltage, with the following characteristics for each stage: initial ascending segment: as displacement increases, force values rise rapidly, indicating the initial hardness of the sample, with faster rates suggesting greater initial hardness; first peak: the force reaches a peak of about 90 N, representing the maximum resistance encountered when the probe first compresses the sample, which can measure the sample’s hardness; descending segment: force values quickly decrease, indicating deformation of the sample and changes in its internal structure, reducing resistance to the probe; second ascending segment: force rises to a second peak of approximately 81 N, allowing comparison with the first peak to assess the stability of hardness under repeated forces; subsequent descending and stable segments: force values rapidly decrease towards 0, indicating significant irreversible deformation or damage to the sample, making it unable to resist the probe clearly. Figure 13b shows the texture test curve of the potato at 15 kV voltage. In the 0–10 mm displacement range, the force values fluctuate with multiple peaks, indicating a complex or uneven internal structure reacting to the probe, with the highest hardness reaching about 111 N. After exceeding 10 mm, the force values rapidly decrease towards 0, remaining extremely low between 10 and 20 mm, indicating that the sample deforms and its structure is destroyed, unable to provide significant resistance to the probe. Near 20 mm, the force value suddenly rises to a peak of 40 N, then quickly decreases again to 0, remaining stable in subsequent displacements. The difference between the second peak and the first is significant, possibly due to changes in the potato’s structure after the first force application, affecting its hardness. Figure 13c–e show the texture curves of the potato under voltages of 20 kV, 25 kV, and 30 kV, respectively. Most stages share similar characteristics with those at 15 kV. At 20 kV, the first peak is approximately 60 N, while at 25 kV it is about 51 N. Near 20 mm, the second peak at 20 kV is 12 N, and at 25 kV it is 31 N. The significant difference between the second and first peaks may be due to changes in the potato sample’s structure after the initial force application, affecting subsequent hardness. At 30 kV, the force values fluctuate with multiple peaks between 0 and 10 mm, reaching up to about 66 N, and a second peak near 27 mm at 61 N. These two peaks are relatively close, indicating that the potato treated with this voltage maintains stable hardness and can retain certain resistance properties under repeated forces.

**Figure 14:**
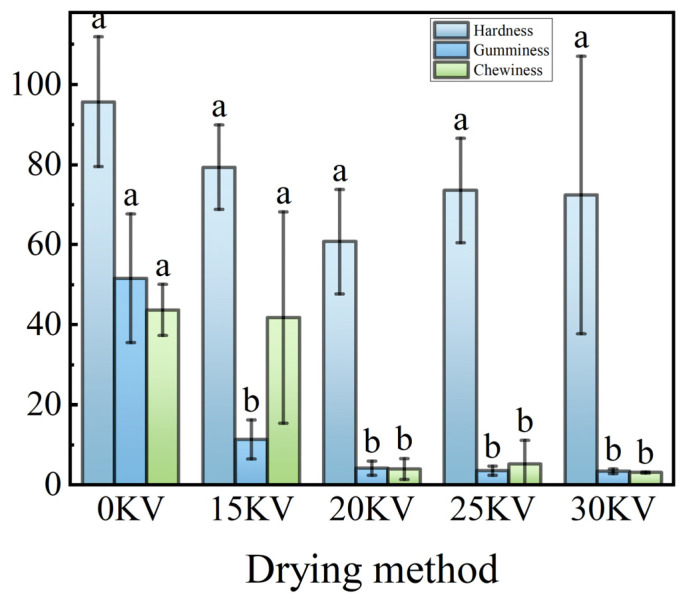
Hardness, gelatinization, and chewiness of potato samples under different drying voltage conditions. The use of different letters indicates significant differences between sample means (*p* < 0.05).

A substantial discrepancy (*p* < 0.05) in the impact of diverse drying methodologies on the textural characteristics of the potato was evident. This variation was closely associated with the intensity of internal binding forces and the configuration of protein in the material [59]. The data in Figure 14 demonstrate that the hardness of potato flakes exhibited a gradient decrease with increasing EHD drying voltage. The phenomenon in question can thus be attributed to the electric field-induced fiber structural remodeling, which triggers the mechanical fracture of fiber bundles, leading to a decrease in macroscopic hardness [60]. It is noteworthy that the low-temperature characteristic of EHD drying (≤60 °C) effectively preserves cellular integrity and avoids protein denaturation caused by conventional thermal drying. This results in a reduction in the molecular spacing of the dehydrated material and the formation of a dense microstructure. The trend of the chewability parameter is analogous to that of hardness, which is due to the fact that chewability is essentially a combination of hardness and elasticity [61].

**Figure 15:**
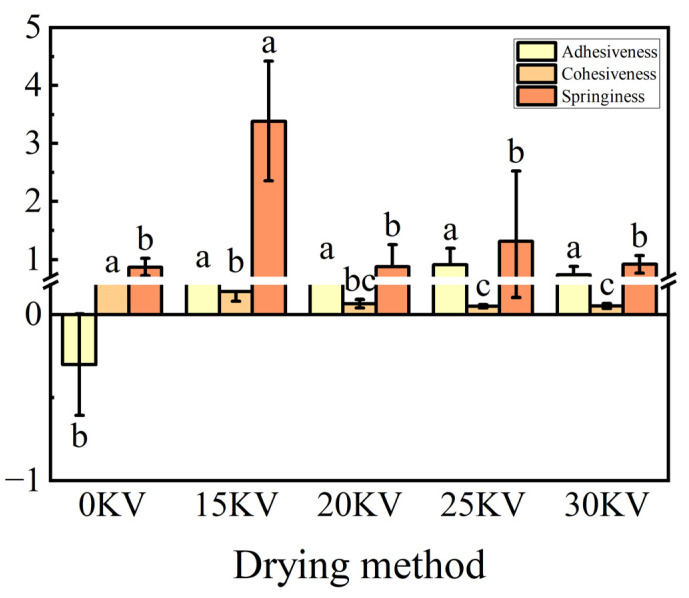
Adhesion, cohesion, and elasticity of potato samples under different drying voltage conditions. Different letters indicate significant differences between sample means (*p* < 0.05).

The cohesion index, conversely, is indicative of the strength of interactions between protein molecules [62]. As demonstrated in Figure 15, the 25 kV treatment group demonstrated the highest adhesion value, suggesting that the protein aggregates formed under these conditions exhibited optimal structural stability. The elasticity parameter exhibited a significant and positive correlation with the protein content [63], with the highest protein content and optimal elasticity observed in the 15 kV treatment group.

The aforementioned results indicated that EHD drying optimized the textural properties by regulating protein conformation and water migration pathways. Among the treatment groups, the 15 kV group exhibited the optimal balance between hardness and elasticity, and its mechanical strength was sufficient to withstand the mechanical stress during transportation, thereby significantly reducing the breakage rate. This finding is consistent with the research conclusion that non-thermal drying technology retains the natural conformation of proteins [64], which provides an important process parameter basis for the development of high value-added dried potato products.

### 3.9. Statistical Analysis

**Figure 16:**
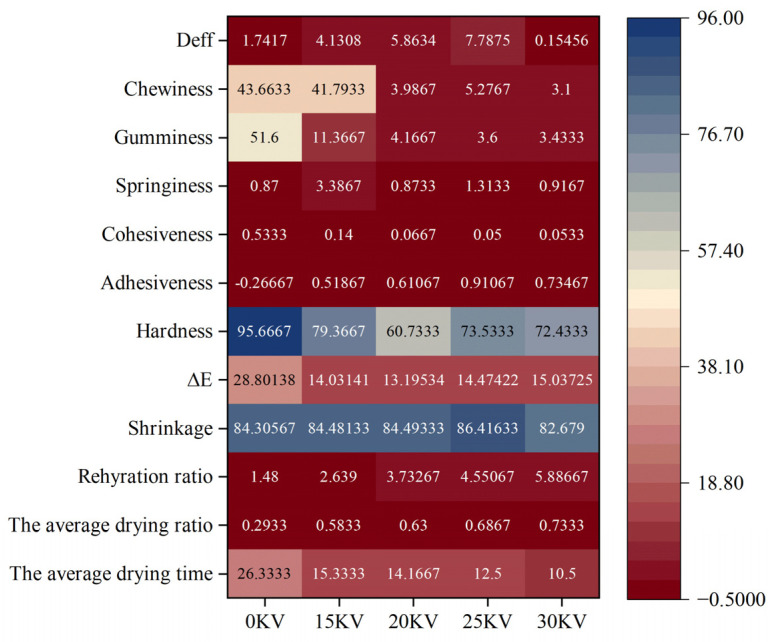
Correlation thermogram of potato drying index under different drying voltage conditions.

In this study, a heat map correlation matrix was constructed between drying parameters and textural properties of the products. Z-score standardization of experimental data was used to create the matrix, as shown in Figure 16. The results showed that the adhesion index was positively correlated with the EHD treatment group and negatively correlated with the 0 kV control group. The 25 kV treatment group exhibited the highest adhesion value, suggesting that the dried products formed under this voltage condition possessed superior soft and glutinous textural properties. It is noteworthy that the 15 kV treatment group demonstrated the strongest positive correlation with the elasticity index. Its modulus of elasticity was three times higher than that of the control group, a characteristic that confers enhanced resistance to mechanical damage during transportation.

**Figure 17:**
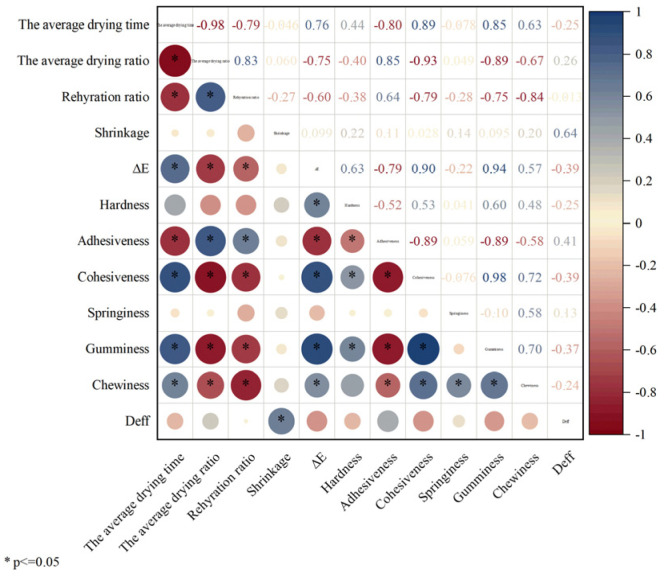
Pearson correlation coefficient matrix of potato under different drying voltage conditions.

Pearson correlation analysis revealed a significant positive correlation between hardness and color difference in Figure 17, suggesting a potential relationship with the degree of Maillard reaction during the drying process. Notably, the average drying rate exhibited a significant negative correlation with color difference, cohesion, and adhesion. This indicates that process parameters must be meticulously controlled to ensure product quality while maintaining drying efficiency. The chewiness index demonstrated a strong positive correlation with adhesion, cohesion, elasticity, and adhesive bonding, suggesting that enhancing chewiness is closely associated with strengthening intercellular bonding strength.

**Figure 18:**
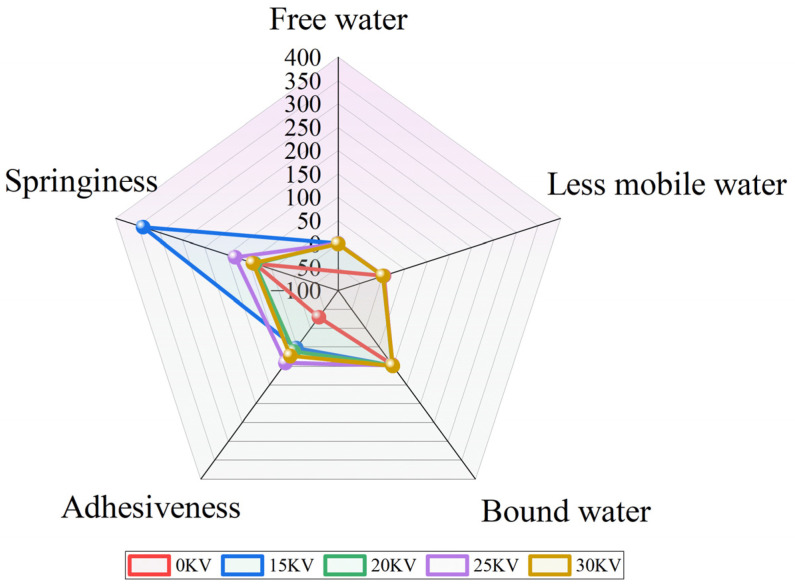
Radar plots of potato LF-NMR versus TPA under different drying voltage conditions. (Bound, immobile, and free water range from 0 to 100, while elasticity and adhesion range from minus 100 to 400; in order to visualize all the parameters in one graph, an image with a free water range greater than 100 is shown for certain scale settings).

The analysis in Figure 18 further corroborates the comprehensive advantages of the 15 kV EHD drying process: the treatment group exhibits superiority over other experimental groups in terms of moisture retention, elasticity, and other key indexes, and the formed dry products possess balanced textural characteristics. It is recommended that in actual production, 25 kV treatment be preferred for soft and glutinous products, while 15 kV is recommended for products that need to take into account the transportation performance.

## 4. Conclusions

This study systematically investigated the influence of different electric field intensities (0–30 kV) on the drying kinetic properties, microstructure evolution, texture characteristics, and moisture distribution of potato. The experiment used a corona discharge device to generate low-temperature plasma, and spectral analysis revealed that the intensity of emission spectra showed an exponential growth trend with increasing voltage, indicating that the generation of energetic active particles was positively correlated with the electric field strength. The EHD drying group exhibited enhanced drying efficiency, color, shrinkage, rehydration performance, and effective moisture diffusion coefficient when compared with the control group. Infrared spectral analysis revealed that the characteristic peak positions at 3256.44 cm^−1^ (O-H stretching vibration), 2925.69 cm^−1^ (C-H stretching vibration), and 1624.95 cm^−1^ (C=O stretching vibration) remained unchanged in the treatment groups, thereby confirming the efficacy of EHD drying in preserving the major carbohydrate and protein components of potato. Amide I spectroscopy analysis revealed that the proportion of protein α-helical structure increased from 12.99% in the control group to 22.04% in the 30 kV group with an increase in electric field intensity, while the proportion of randomly curled structure decreased from 15.07% to 12.88%. These findings suggest that the high-voltage electric field elevated the molecular ordering by altering the secondary structure of proteins. Furthermore, the results of scanning electron microscopy observations revealed that potato cells in the EHD-treated group exhibited characteristics of directional fracture of fibrous structure, and the starch granules were completely exposed due to the destruction of the cell wall, forming a porous network structure. The findings of the present study demonstrate that low-field nuclear magnetic resonance (LF-NMR) results indicate that the EHD-treated group exhibited a higher capacity for bound water retention, thereby suggesting that this technique possesses significant advantages in maintaining cellular structural integrity. Furthermore, the texture analysis revealed that the interaction of drying temperature and time exerted a substantial effect on product cohesion and adhesion. The adhesion of potato reached its peak under 25 kV treatment conditions, a finding that lends itself to the development of soft and glutinous food products. Meanwhile, the 15 kV process parameter ensured drying efficiency while concurrently increasing the elastic modulus of the product to the optimal level, a development that led to a significant reduction in the transportation breakage rate. In sum, the findings of this study furnish a novel technical solution for the potato drying and deep processing industry.

## Figures and Tables

**Table 1:** Effective diffusion coefficients of internal moisture in potato samples at different drying voltages.

Voltage	Linear Model	R^2^	D_eff_ (m^2^/s)	F	*p*
0 kV	lnMR = −1.91 × 10^−5^t + 0.02336	0.98958	1.7417 × 10^−11^	120.37	2.2 × 10^−9^
15 kV	lnMR = −4.53 × 10^−5^t + 0.12226	0.97550	4.1308 × 10^−11^	128.34	1.5 × 10^−9^
20 kV	lnMR = −6.43 × 10^−5^t + 0.22602	0.95872	5.8634 × 10^−11^	130.20	1.2 × 10^−9^
25 kV	lnMR = −8.54 × 10^−5^t + 0.33431	0.90363	7.7875 × 10^−11^	125.84	2.0 × 10^−9^
30 kV	lnMR = −1.69 × 10^−4^t + 0.84121	0.84784	1.5456 × 10^−10^	130.76	1.1 × 10^−9^

**Table 2:** Variation of color difference of potato samples under different drying voltage conditions.

Voltage	L_1_	a_1_	b_1_	∆E	C	Whiteness	h°
Fresh	38.20 ± 0.90 ^a^	5.66 ± 0.33 ^a^	14.89 ± 0.40 ^a^	—	—	—	—
0 kV	16.90 ± 6.52 ^c^	5.80 ± 5.93 ^a^	6.38 ± 3.18 ^b^	28.80 ± 7.78 ^a^	8.96 ± 6.04 ^b^	16.29 ± 6.87 ^b^	0.93 ± 0.37 ^a^
15 kV	26.49 ± 1.65 ^b^	6.74 ± 1.79 ^a^	14.08 ± 1.53 ^a^	14.03 ± 1.79 ^b^	15.65 ± 1.99 ^a^	24.82 ± 1.21 ^a^	1.12 ± 0.07 ^a^
20 kV	26.52 ± 1.11 ^b^	4.83 ± 0.87 ^a^	12.10 ± 0.28 ^a^	13.19 ± 1.37 ^b^	13.05 ± 0.57 ^ab^	25.37 ± 0.99 ^a^	1.19 ± 0.05 ^a^
25 kV	25.16 ± 0.58 ^b^	5.67 ± 0.95 ^a^	14.56 ± 0.96 ^a^	14.47 ± 0.42 ^b^	15.64 ± 0.86 ^a^	23.54 ± 0.38 ^a^	1.19 ± 0.06 ^a^
30 kV	24.65 ± 1.57 ^b^	4.82 ± 3.86 ^a^	15.64 ± 2.52 ^a^	15.03 ± 2.51 ^b^	16.68 ± 2.28 ^a^	22.79 ± 1.03 ^a^	1.27 ± 0.24 ^a^

Different letters indicate significant differences in sample means (*p* < 0.05). L_1_: luminance value; a_1_: red value; b_1_: yellow value; ∆E: total color difference.

**Table 3:** Protein secondary structure of potato samples under different drying voltage conditions.

Conditions	Random Coil	α-Helix	β-Turn	β-Antiparallel	β-Sheet
0 kV	15.07%	12.99%	27.19%	2.82%	41.93%
15 kV	12.77%	10.02%	22.23%	1.70%	53.28%
20 kV	11.99%	11.50%	24.14%	2.21%	50.16%
25 kV	12.76%	10.87%	22.65%	3.58%	50.14%
30 kV	12.88%	22.04%	14.95%	6.16%	43.97%

## Data Availability

The original contributions presented in this study are included in the article. Further inquiries can be directed to the corresponding author.
